# Editorial: DNA Damage and Inflammation under Stress

**DOI:** 10.3389/fgene.2017.00152

**Published:** 2017-10-17

**Authors:** Alexandros G. Georgakilas, Athanassios Kotsinas

**Affiliations:** ^1^DNA Damage Laboratory, Physics Department, School of Applied Mathematical and Physical Sciences, National Technical University of Athens, Athens, Greece; ^2^Molecular Carcinogenesis Group, Department of Histology and Embryology, School of Medicine, National and Kapodistrian University of Athens, Athens, Greece

**Keywords:** DNA damage, inflammation, systemic effects, cellular stress, immune system

In this Research Topic, we have explored the synergistic effects and mechanisms between DNA damage induction and inflammatory response under a variety of cellular stresses like radiations, replication stress, environmental chemicals, bacteria etc. In general, we believe that priority in research must be given to studies following a holistic approach and at the organism level from plants, flies, worms to mouse, and humans. We consider this to be a genuine example of a Systems Biology application in life. Several lines of evidence suggest that DNA-damage especially the one resistant to repair can cause immune reaction and inflammation, and is linked to cancer development and other pathologies (Ermolaeva et al., 2013; Karakasilioti et al., 2013). On the other hand, it is generally accepted that chronic inflammation can cause DNA persistent damage and act as a driver for pathophysiological phenotypes like cancer (Pateras et al., 2015).

The present Research Topic nicely represents the different approaches and current understanding in the association between DNA damage, inflammatory and immune response under different types of stress. These approaches include cell biology, biochemistry, genetics, bioinformatics, as well as computational and experimental evidence. Scientists from all these fields work effectively together to unravel the mechanisms of how a type of stress in the organism can result to the induction of a chronic state of inflammation and immune system triggering, primarily, with the DNA damage acting as the mediator.

In this Research Topic we had contributions relative to the above field and groups working in all of the above areas and model organisms linking DNA damage, inflammatory and innate immune response, and a variety of systemic effects associated with the above phenomena. Special interest was given by all contributing groups to the role of DNA damage response (DDR) genes and their link with inflammatory proteins, underlying molecular mechanisms justifying the system-level analysis of systemic action, computational and bioinformatic approaches for better description of real biological systems and integration of current empirical or theoretical knowledge.

Specifically, in the review article of Spanou et al. the role of genetic variability as a regulator of TLR4 and NOD signaling in response to bacterial driven DNA Damage Response (DDR) and Inflammation is discussed with a focus on the gastrointestinal (GI) Tract. The fundamental role of human Toll-like receptors (TLRs) and NOD-like receptors (NLRs), the two most studied pathogen recognition receptors (PRRs), is also explored as one of the protection mechanisms against pathogens and excessive tissue injury. On the same direction, in a mini-review article by Kalisperati et al. the most current picture of the molecular pathways involved in DNA damage and DDR activation during *H. pylori* infection and gastric tumorigenesis are discussed. As well-known, *Helicobacter pylori* (*H. pylori*) is a Gram negative bacterium that colonizes the stomach of almost half of the human population, therefore the interest on the current subject is high.

Then moving to the DDR-related mechanisms of organism homeostasis, in an analytical review, Eliopoulos et al. uncover the mechanisms by which autophagy and DDR act as partners regulating cellular and organismal homeostasis. In this review, the authors provide novel insights into the pathobiology of conditions associated with accumulation of DNA damage, including cancer and aging, and original concepts for the development of improved therapeutic strategies against these pathologies. Aging is certainly a process associated with DDR and inflammation (Karakasilioti et al., 2013). In a mini-review article, Ioannidou et al. nicely cover the most-current status of the knowledge on how DNA damage-driven inflammation in the context of tissue-specific pathology can act as a driver for disease (aging) progression. Last but certainly not least, Nakad and Schumacher review the very important and recent advances on the understanding of the role(s) of DDR in activating immune signaling. They provide convincing evidence gained into (i) which molecular and cellular pathways of DDR activate immune signaling, (ii) how DNA damage drives chronic inflammation, and (iii) how chronic inflammation causes DNA damage and pathology in humans.

We are grateful to all contributors of this Research Topic. In total, twenty-five different authors from three (3) different countries contributed with analytical and current review articles. Furthermore, we thank the reviewers which helped us and the authors to create an interesting and high-quality Research Topic.

We hope that you enjoy reading this Research Topic as much as we have enjoyed editing it and it will further promote the field under a systems biology perspective.

## Author contributions

AG wrote the initial draft of the editorial; both authors revised the manuscript and approved the final version of it.

### Conflict of interest statement

The authors declare that the research was conducted in the absence of any commercial or financial relationships that could be construed as a potential conflict of interest.
